# Magnitude, determinants, and adverse outcomes of unintended pregnancy among pregnant mothers in low- and middle-income countries: An umbrella review of systematic review and meta-analysis

**DOI:** 10.7189/jogh.14.04253

**Published:** 2024-12-13

**Authors:** Gizachew Yilak, Tegene Atamenta Kitaw, Biruk Beletew Abate, Alemu Birara Zemariam, Addis Wondmagegn Alamaw, Eyob Shitie Lake, Mulat Ayele, Alemayehu Sayih Belay, Addisu Getie, Befkad Derese Tilahun

**Affiliations:** 1 Department of Nursing, College of Health Sciences, Woldia University, Woldia, Ethiopia; 2 Department of Midwifery, College of Health Sciences, Woldia University, Woldia, Ethiopia; 3 Department of Nursing, College of Health Sciences, Wolkite University, Wolkite, Ethiopia; 4 Department of Nursing, College of Health Sciences, Debre Markos University, Debre Markos, Ethiopia

## Abstract

**Background:**

To date, findings from systematic reviews and meta-analyses on unintended pregnancies in low-income and middle-income countries (LMICs) are inconsistent, posing challenges for preventive efforts. Therefore, the aim of this study is to determine the magnitude, determinants, and adverse outcomes of unintended pregnancy among pregnant mothers in LMICs: an umbrella review of systematic review and meta-analysis.

**Methods:**

PubMed, Scopus, Science Direct, Web of Science, as well as databases specific to systematic reviews, such as the Cochrane Database, have investigated the magnitude, risk factors, and adverse outcomes of unintended pregnancy in LMICs. The methodological quality of the included studies was assessed using the Assessment of Multiple Systematic Reviews (AMSTAR) tool. The estimates from the included studies regarding the magnitude and predictors of unintended pregnancy were then pooled and summarised using random-effects meta-analysis models.

**Results:**

We included 13 systematic review and meta-analysis (SRM) studies involving 1 446 122 women. The summary estimate for the magnitude of unintended pregnancy was 28.38% (95% CI = 23.06–33.7%, *I*^2^ = 100%). From the umbrella review, the reported factors and complications of statistical significance were as follows: maternal illiteracy (AOR = 3.79; 95% CI = 1.36–8.94), being unmarried (AOR = 12.98; 95% CI = 1.88–27.85), lack of communication with the husband about family planning (AOR = 3.43; 95% CI = 1.68–5.19), inability to attend antenatal care (AOR = 1.4; 95% CI = 0.62–2.17), never using family planning (AOR = 1.4; 95% CI = 0.62–2.17), maternal depression (AOR = 1.72; 95% CI = 0.81–2.64), stunting (AOR = 1.76; 95% CI = 1.25–2.48), and parity 3.83 (AOR = 1.3; 95% CI = 1.3–11.3).

**Conclusions:**

The pooled magnitude of unintended pregnancies in LMICs was high. Therefore, it is crucial to integrate family planning and maternal health care services to prevent unintended pregnancy. Additionally, interventions targeting rural, unmarried, less-educated, and adolescent women are important for preventing unintended pregnancies in LMICs.

An unintended pregnancy refers to a situation in which a woman becomes pregnant either before they plan or when they have no desire to become pregnant [1]. It is a worldwide public health issue affecting sexually active women [2]. Moreover, it has significant health, economic, and social repercussions for women, their children, and their families [3]. This represents a significant global public health challenge that has profound implications for the well-being of mothers, their offspring, families, communities, and nations [4,5].

From 2015 to 2019, there were approximately 121 million pregnancies that were not intended on a global scale, resulting in an average of 64 unintended pregnancies per 1000 women aged 15–49 years [6]. In low and middle-income countries (LMICs), approximately 74 million women experienced unintended pregnancies, leading to an annual occurrence of approximately 25 million unsafe abortions and 47 thousand maternal deaths [7]. In sub-Saharan Africa, the prevalence of unintended pregnancies is ranged from 10.8 to 54.27% [8-10]. Although the number of unintended pregnancies in the developing world is difficult to ascertain, its scope can be surmised by the following statistics. The World Health Organization (WHO) estimates that there are 70 000 maternal deaths each year from complications of unsafe, illegal abortions and that approximately 585 000 women die every year from complications related to pregnancy and childbirth [9-11].

Mothers who experience unintended pregnancies face various severe complications including induced abortions, which can result in maternal mortality [11]. Additionally, they were more prone to higher crime rates, parenting and family stress, reduced work productivity, lower academic accomplishments, a high risk of poor physical and mental health, inadequate self-care practices, and depression during pregnancy [11,12]. Furthermore, mothers with unintended pregnancies are more likely to show negligence towards their pregnancy, leading to delayed initiation of antenatal care and reduced utilisation of delivery services [2,9,11-14].

Unintended pregnancies and their complications are a problem in LMICs that affects families, women, and the community. They typically result in intentional abortion or even the death of the mother. As a result, lowering unintended pregnancies leads to lower rates of maternal mortality as well as lower hospital expenses and workload. Additionally, it can ensure the pattern of population increase, the level of couples' welfare, and the social and economic advancement of society [15-17].

To address the challenges posed by unintended pregnancies in LMICs, several interventions have been implemented to improve the contraceptive prevalence rate (CPR) and reduce the unmet need for contraception. To assess the progress made in this regard, it is crucial to identify the level of unintended pregnancy and its associated factors in LMICs [18].

In LMICs, several meta-analyses, systematic reviews, and primary research studies have assessed the prevalence of unintended pregnancy and its associated factors from different perspectives and levels, such as national and local perspectives. However, there are inconsistent reports that pose challenges for health care programmes and clinical decision making. To address these inconsistent findings related to unintended pregnancy, it is crucial to gather evidence on the extent and factors that contribute to unintended pregnancy. Therefore, the primary objective of this umbrella review was to provide a comprehensive summary of the combined estimates of unintended pregnancy, determinants, and adverse outcomes in LMICs.

## METHODS

This umbrella review was conducted in accordance with the methodology of an umbrella review of multiple systematic reviews [19]. It was undertaken through a systematic synthesis of eligible SRM reports on unintended pregnancy, its predictors, and adverse outcomes in LMICs.

### Search strategy

Five international online databases, PubMed, Scopus, Science Direct, Web of Science, and databases specific to systematic reviews, such as the Cochrane Database of Systematic Reviews and the Database of Abstracts of Reviews of Effects, were searched for SRM studies on unintended pregnancy, its predictors, and complications in LMICs. To access relevant data regarding unintended pregnancy, a comprehensive search was conducted using the aforementioned databases and the adapted PICO questions. These questions were developed from search keywords and/or Medical Subject Headings (MeSH), which were combined using the 'OR' and 'AND' Boolean operators

a) Population: adolescents, youths, and women of reproductive age.

b) Outcome: unintended pregnancy, determinants, predictors, associated factors, correlates, risk factors, adverse effects of unintended pregnancy, complications of unintended pregnancy, and consequences of unintended pregnancy.

c) Study design: systematic review, meta-analysis of observational studies

d) Setting (context): LMIC

Both published and unpublished studies were searched for in this umbrella review. A literature search was conducted from 28 October 2023, until 14 December 2023. Two independent researchers performed the literature search and any discrepancies were resolved through discussion and consensus with the remaining authors. A sample literature search strategy, specifically the PubMed search strategy, was developed using a combination of Medical Subject Headings (MeSH) terms and free text.

### Eligibility criteria

#### Inclusion criteria

Publications published between January 2010 and August 2023 was eligible for inclusion in the study. The time restriction aimed to ensure that the findings reflected, or were related to, the current state of women's health in these countries. The following predefined criteria were considered for a study to be regarded as a systematic review or meta-analysis:

a) Presentation of a defined literature search strategy

b) Appraisal of the included studies using a relevant tool

c) Adherence to a standard approach for pooling studies and providing summary estimates for all types of women of reproductive age.

#### Exclusion criteria

Studies were excluded if they met any of the following criteria:

a) No report on the prevalence or determinants of unintended pregnancy or complications of unintended pregnancy

b) Narrative reviews editorials, correspondence, abstracts, and methodological studies.

Furthermore, literature reviews that lacked a defined research question, search strategy, or article selection process were excluded.

### Data extraction

Data from the included SRM studies were extracted using a standardised data abstraction form developed in an Excel spread sheet. For each SRM study, the following data were extracted:

a) Identification data (first author's last name and publication year)

b) Review aim

c) Prevalence or proportion of unintended pregnancy

d) Complications of unintended pregnancy

e) Risk factors for unintended pregnancy

f) Odds ratio or relative risk with 95% confidence intervals for the risk factors of unintended pregnancy

g) Number of primary studies included within each SRM study and their respective design type

h) Total number of sample sizes included

i) Publication bias assessment methods and scores

j) Quality assessment methods and scores

k) Data synthesis methods (random- or fixed-effects model)

l) The authors' main conclusion of the SRM study.

### Risk of bias assessment

All the included studies were critically appraised to assess the validity and scoring of the results. We used the Assessment of Multiple Systematic Reviews (AMSTAR) tool to ensure the methodological and evidence quality of the included SRM studies [18,20].

### Data synthesis

Both narrative (qualitative) and quantitative approaches were used to summarise the estimates of the included SRM studies. In cases where two or more estimates were provided for the magnitude, associated factors, and complications of unintended pregnancy, the range of these estimates was presented and a summary (pooled) estimate was calculated. The choice of meta-analysis model was guided by the level of heterogeneity between studies, which was assessed using Higgins' *I*^2^ statistics [20]. The Der Simonian-Laird random-effects model was employed to pool (summarise) magnitude estimates owing to the high level of between-study heterogeneity [20]. Assessment of publication bias was not feasible due to the inclusion of only four studies. A minimum of 10 studies are typically required to evaluate publication bias [19,21]. Quantitative analyses were performed using the STATA version 17.0. A summary list of the predictors of unintended pregnancies and their respective odds ratios was prepared.

### Ethical consideration

In this study, no study participants' consent or ethical approval was required because the study was conducted based on data extracted from SRM studies.

## RESULTS

### Literature search findings

**Figure 1:**
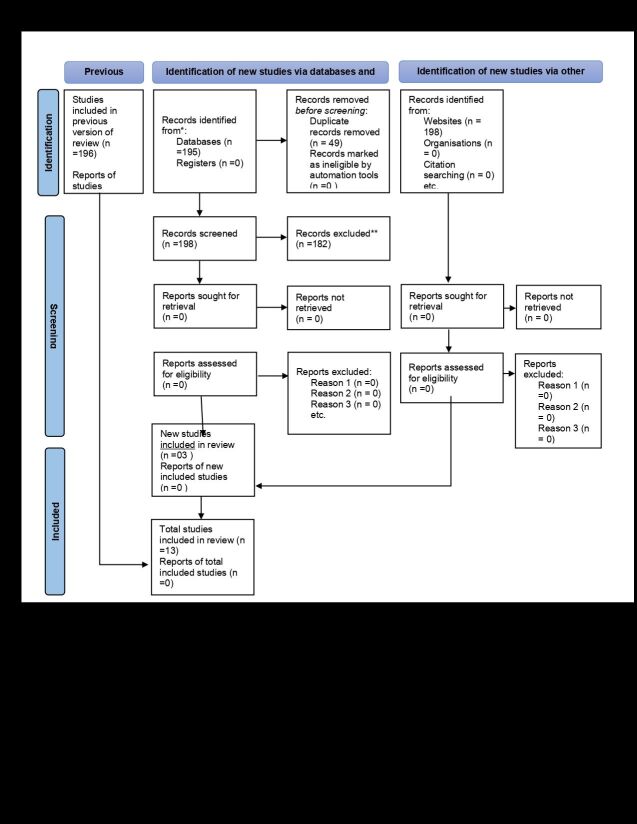
PRISMA flowchart of selection for an umbrella review of systematic reviews and meta-analyses on unintended pregnancy and determinants and adverse outcomes among pregnant mothers in low- and middle-income countries.

The database search yielded 198 articles, of which 49 remained after removing duplicates. Of these 49 articles, 35 were excluded during title and abstract screening because they did not pertain to SRM studies, which was the specific focus of this study on unintended pregnancy. After conducting a full-text review of the remaining 14 articles, one SRM study was excluded owing to its failure to address the required outcome. Therefore, 13 SRM studies were included in the final analysis [9,22–34], This umbrella review includes 13 SRM studies. The study selection and screening process are shown in **Figure 1**.

### Characteristics of the included review studies

This umbrella review encompassed observational studies and clinical trials, specifically 21 cohort studies, three case-control studies, 13 clinical trials, and 286 cross-sectional studies. These studies, totalling 323, involved a combined sample size of 1 446 122 women. The number of systematic reviews and meta-analyses ranged from 12 (lowest) [32] to 55.9 (highest) [23]. The sample size per meta-analysis ranged from 820 (lowest) [31] to 474 901 (highest) [25]. Eight SRM studies [9,22,23,26,28,30,31,33,34] were published between 2013 and 2018 and the remaining SRM studies [25,27,29,32] were published between 2019 and 2023. According to the included eight SRM studies, the reported estimate of the magnitude of unintended pregnancy was 28.071 (95% CI = 22.818–33.324%), *I*^2^ = 100% (**Table 1**).

**Table 1:** General characteristics of included systematic reviews and meta-analyses, databases, and search techniques used for identifying primary studies

Author (year)	Review aim	Search strategy	Included studies	Sample size	Risk of bias	Reported prevalence	Authors' conclusions	AMSTAR Quality
Muluneh Alene et al. (2019) [35]	Prevalence and determinants of unintended pregnancy in Ethiopia	Google Scholar, PubMed, Science Direct, Web of Science, CINAHL, and Cochrane Library), and national (Ethiopian Journal of Public Health and Nutrition) electronic databases	Community based cross-sectional studies = 12. Institution based cross-sectional studies = 13.	23 536	Clear quality appraisal of the studies has been started	28	A high prevalence of unintended pregnancy was observed. Lack of spousal communication, never using family planning, maternal education, and household wealth level were significantly associated with an unintended pregnancy.	13
Achenef Asmamaw Muche et al. (2018) [22]	Prevalence and determinants of risky sexual practice in Ethiopia	PubMed, Google scholar, CINAHL, and African Journals Online were included in the review.	Cross-sectional studies = 31	43 695	Clear quality appraisal of the studies has been started	18.8	The prevalence of risky sexual practice was high in Ethiopia. Being male, substance use, peer pressure and viewing pornographic materials were found to be associated with risky sexual practices.	12
Tesfaye Regassa Feyissa et al. (2018) [23]	Unintended Pregnancy in Women Living with HIV in sub-Saharan Africa:	MEDLINE, PubMed, Embase, PsychINFO, Scopus and CINAHL, with additional articles searched in Google Scholar and the African Index Medicus through the World Health Organization database.	Cross-sectional studies = 151. Prospective cohort studies = 2.	4662	Clear quality appraisal of the studies has been started	55.9	The current evidence indicates that a substantial number of pregnancies in WLHIV in sub-Saharan Africa were unintended, being unwanted or mistimed. However, this conclusion is mainly based on cross-sectional surveys.	14
Frances H Ampt et al. (2018) [24]	Incidence of unintended pregnancy among female sex workers in low-income and middle-income countries	MEDLINE, Embase, PsychINFO and Popline was undertaken to identify relevant peer-reviewed articles and Web of Science and Proquest databases	Prospective cohor = 13. Random control trial studies 12.	13 521	Clear quality appraisal of the studies has been started	26.8	Access to family planning, particularly long-acting reversible contraceptives, may be improved by better targeting of FSWs through mobile outreach71 and integration with existing FSW-specific HIV prevention services, and by careful training of health workers and community workers in contraceptive counselling and follow-up.	12
Annisa Lidra Maribeth et al. (2019) [30]	The association of unintended pregnancy with stunting on children under five years old	Scopus, Google Scholar, Pubmed from January 2015 to May 2018.	Prospective cohort = 2. Case-control studies = 3.	32 355	Clear quality appraisal of the studies has been started.	27	Unintended pregnancy should be decreased immediately because the decline in the number of unintended pregnancies not only prevents the occurrence of stunting but also can improve the quality of life of the mother and child.	14
Amanuel Alemu Abajobir et al. (2016) [31]	The association between unintended pregnancy and perinatal depression	PubMed, EMBASE, PsycINFO and Google Scholar) and hand searches of reference lists of included.	Prospective cohort = 4. Cross-sectional studies = 5. Random control trial = 1.	820	Clear quality appraisal of the studies has been started.	28	The prevalence of perinatal depression is 2-fold in women with unintended pregnancy. Perinatal care settings may screen pregnancy intention and depression of women backed by integrating family planning and mental health services.	14
Kindie Mitiku Kebede et al. (2021) [32]	Prevalence and determinants of unintended pregnancy in Ethiopia	Medline/PubMed, Cochrane library, Cumulated Index to Nursing and Allied Health Literature (CINAHL), Google scholar and African journal online. online library of academic institutions in Ethiopia using the Google search Engine.	Cross-sectional = 25	23 030	Clear quality appraisal of the studies has been started.	12	Unintended pregnancy in Ethiopia was high. Empowering women and ensuring the accessibility of quality family planning services. Interventions that target rural, poor, unmarried, multiparous, less-educated, and adolescent women are also important to avert untended pregnancy.	13
Mahmood et al. (2013) [34]	Prevalence of unwanted pregnancy in Iran: a systematic review and meta-analysis	Medline/PubMed, Cochrane library, Cumulated Index to Nursing and Allied Health Literature (CINAHL), Google scholar	Cross-sectional = 49	43 061		30.6	One-third of pregnancies in Iran are unwanted. Therefore‚ appropriate policies on the education, proper pregnancy age, using contraceptive methods, men's role in family planning programmes and quality promotion	13

### Methodological quality of the included SRM studies

The methodological quality of the included SRM studies evaluated using the AMSTAR tool [36]. Quality scoring was conducted on a 16-point scale ranging from 12 to 14, with a mean score of 13. Among the AMSTAR criteria assessed, the most frequently satisfied criteria across the review studies were a priori design, duplicate study selection and data extraction, appropriateness of the methods used to combine the study findings, and the disclosure of conflicts of interest. In contrast, the AMSTAR criteria that were less frequently satisfied included search comprehensiveness, provision of included and excluded studies, and appropriate utilisation of the scientific quality of the included studies in formulating conclusions (**Table 2**). The AMSTAR criteria that were assessed included:

**Table 2:** Methodological quality of the included studies based on the AMSTAR tool*

Author, year	Q1	Q2	Q3	Q4	Q5	Q6	Q7	Q8	Q9	Q10	Q11	Q12	Q13	Q14	Q15	Q16	Total
Muluneh et al., (2019)	yes	yes	yes	yes	yes	no	yes	yes	yes	no	yes	yes	yes	no	yes	yes	13
Achenef et al., (2018)	yes	yes	no	yes	yes	no	yes	yes	yes	no	yes	no	yes	yes	yes	yes	12
Tesfaye et al., (2018)	yes	yes	yes	yes	yes	no	yes	yes	yes	yes	yes	yes	yes	yes	no	yes	14
Frances et al., (2018)	yes	no	yes	yes	yes	no	yes	yes	yes	no	yes	yes	yes	yes	no	yes	12
Nuruzzaman et al., (2019)	yes	yes	yes	yes	yes	no	yes	yes	yes	no	yes	yes	yes	no	yes	yes	13
Yohannes et al. (2013)	yes	yes	yes	yes	yes	no	yes	yes	yes	yes	yes	yes	no	yes	yes	yes	14
Tadesse et al., (2020)	yes	yes	yes	yes	yes	yes	yes	yes	yes	no	yes	yes	yes	no	yes	yes	14
Amie et al., (2015)	yes	yes	yes	no	yes	no	yes	yes	yes	no	yes	yes	no	yes	yes	yes	12
Heidi et al., (2022) [29]	yes	yes	yes	yes	yes	no	yes	yes	yes	no	yes	yes	yes	no	yes	yes	13
Annisa et al., (2018)	yes	yes	yes	yes	yes	no	yes	yes	yes	yes	yes	yes	yes	yes	yes	yes	14
Amanuel et al., (2016)	yes	yes	yes	yes	yes	no	yes	yes	yes	yes	yes	yes	yes	no	yes	yes	14
Kindie et al., (2021)	yes	yes	yes	yes	yes	no	yes	no	yes	yes	yes	yes	yes	yes	no	yes	13
Mahmood et al. (2014)	yes	yes	yes	yes	yes	no	yes	no	yes	yes	yes	yes	yes	no	yes	yes	13

*AMSTAR is a measurement tool to assess systematic reviews.

Q1: a priori design

Q2: duplicate study selection and data extraction

Q3: search comprehensiveness

Q4: inclusion of grey literature

Q5: included and excluded studies provided

Q6: characteristics of the included studies provided

Q7: scientific quality of the primary studies assessed and documented

Q8: scientific quality of the included studies used appropriately in formulating conclusions

Q9: appropriateness of methods used to combine studies' findings

Q10: likelihood of publication bias was assessed

Q11: assess the potential impact of individual studies

Q12: account for RoB in individual studies when interpreting the result

Q13: satisfactory explanation for discussion

Q14: assessing adequate investigation of publication bias

Q15: did the authors report any potential sources of conflict of interest

Q16: conflict of interest – potential sources of support were clearly acknowledged in both the systematic review and the included studies.

### Meta-analysis

#### Prevalence of unintended pregnancy

**Figure 2:**
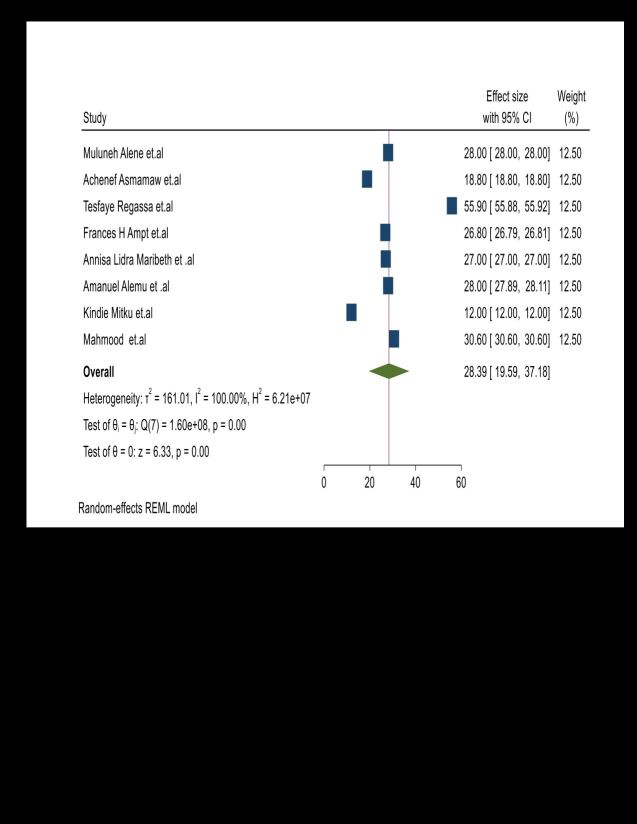
The pooled magnitude of unintended pregnancy among pregnant mothers in low and middle developing country.

From umbrella review of the eight SRM studies [22-24,30-32,34,37], the summary (pooled) magnitude of unintended pregnancy was 28.38% (95% CI = 23.06–33.7%, *I*^2^ = 100%). However, the systematic review findings ranged from 12% [32] to 55.9% [23] (**Figure 2**). We have also checked publication bias and a funnel plot showed symmetrical distribution. Egger’s regression test *P*-value was 0.57, which indicated the absence of publication bias (Figures S2–3 in the **Online Supplementary Document**).

#### Risk factors and complication of unintended pregnancy

Thirteen SRM studies [8,22-29,31,32,34,37] investigated various factors associated with unintended pregnancy and its complications. These studies reported factors such as maternal educational status, lack of communication with the husband about family planning, never using family planning methods, parity, marital status, residence, inability to attend antenatal care (ANC), lower utilisation of delivery health care services, institutional delivery care services, maternal depression, abortion, stunting, and low birth weight. In this umbrella review, the aforementioned factors are categorised into sociodemographic, obstetric, and neonatal factors, as described in the subsequent sections.

#### Sociodemographic factors

**Figure 3:**
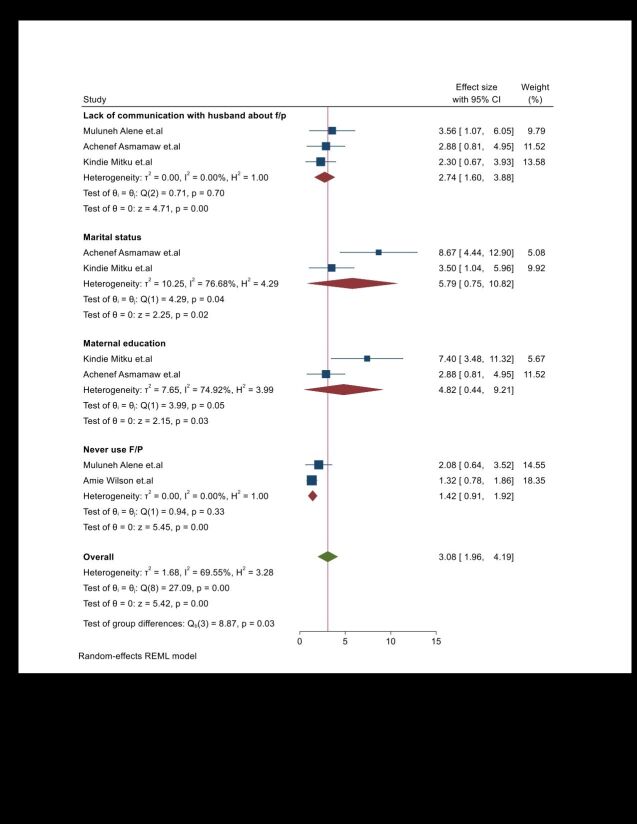
Umbrella review about the pooled effects of maternal socio-demographic and reproductive risk factors on unintended pregnancy. CI – confidence interval

This umbrella review demonstrated the statistical significance of maternal educational status and parity in relation to the burden of unintended pregnancies. According to these reports, mothers who were unable to read and write had a 4-fold increased likelihood adjusted odds ratio ((AOR) = 3.79; 95% CI = 1.355–8.94)) of experiencing unintended pregnancies compared to those who were able to read and write (**Figure 3**). Additionally, neonates born to primiparous mothers had an almost 4-fold increased likelihood (AOR = 3.83; 95% CI = 1.3–11.3) of being associated with an unintended pregnancy compared with neonates born to multiparous mothers. Furthermore, this umbrella review found statistical significance regarding the burden of unintended pregnancy among unmarried women. According to these reports, unmarried women were nearly 12 times more likely (AOR = 12.98; 95% CI = 1.88–27.84) to experience unintended pregnancies than married women (**Figure 3**).

#### Maternal obstetrics and reproductive factors

**Figure 4:**
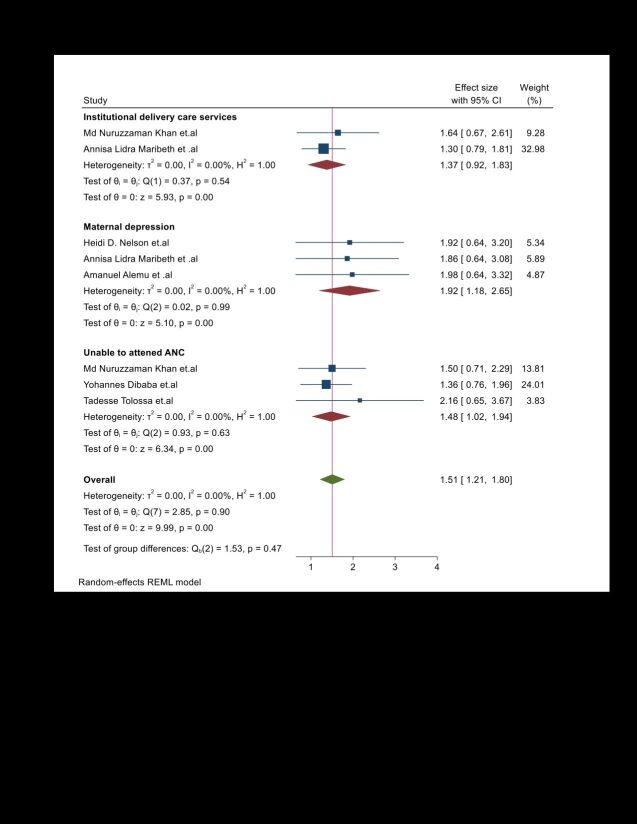
Umbrella review about the pooled effects of maternal obstetrics risk factors on unintended pregnancy. CI – confidence interval

This umbrella review demonstrated the statistical significance of the lack of communication with the husband regarding family planning in relation to the burden of unintended pregnancy. According to these reports, mothers who lacked communication with their husbands about family planning were nearly three times more likely (AOR = 3.43; 95% CI = 1.67–5.18) to have unintended pregnancies compared to those who had communication with their husbands about family planning (**Figure 4**). Additionally, this umbrella review found statistical significance regarding the burden of unintended pregnancy related to the lack of institutional delivery care services. According to these reports, mothers who lacked access to institutional delivery care services were approximately 1.5 times (AOR = 1.5; 95% CI = 0.65–2.65) more likely to experience unintended pregnancies compared to those who had access to such services.

This umbrella review demonstrated the statistical significance of the inability to attend antenatal care in relation to the burden of an unintended pregnancy. According to these reports, mothers who were unable to attend antenatal care were almost 1.4 times (AOR = 1.4; 95% CI = 0.89–1.92) more likely to have unintended pregnancies compared to those who were able to attend antenatal care (**Figure 4**).

Furthermore, this umbrella review found a statistically significant association between the burden of unintended pregnancy and never using family planning. According to these reports, mothers who never used family planning methods were approximately 1.39 times (AOR = 1.395; 95% CI = 0.617–2.173) more likely to experience unintended pregnancies than those who used family planning methods (**Figure 4**).

#### Adverse effect of unintended pregnancy

This umbrella review demonstrated the statistical significance of unintended pregnancies and maternal depression. According to these reports, mothers who experienced unintended pregnancies were almost 1.72 times (AOR = 1.72; 95% CI = 0.806–2.639) more likely to have maternal depression than mothers who had intended pregnancies (**Figure 4**). Furthermore, this umbrella review reported a statistically significant association between unintended pregnancies and abortion. According to these reports, mothers with unintended pregnancies are more likely to have abortions than those with intended pregnancies.

Moreover, this umbrella review revealed a statistically significant relationship between unintended pregnancy, low birth weight, and stunting. According to these reports, mothers who experienced unintended pregnancies were 1.76 times more likely to have children with stunting than those who experienced intended pregnancies. Additionally, those with unintended pregnancies were more likely to have a lower birth weight than those with intended pregnancies were.

## DISCUSSION

To date, there have been thirteen SRM reports that have focused on unintended pregnancies in LMICs. Umbrella review studies provide a high level of evidence for decision-making in health programmes. Therefore, an umbrella review was conducted to consolidate the findings of the 13 SRM studies in a single document. An umbrella review revealed that unintended pregnancy is highly prevalent and a significant public health concern in LMICs. This review also identified various factors related to maternal socio-demography, antenatal and intra-partum periods, and neonatal characteristics that were statistically significant in determining the burden of unintended pregnancies in these countries.

An umbrella review of the eight SRM studies included in the analysis of the burden of unintended pregnancy in LMIC revealed a summary estimate of 28.39% (95% CI = 23.06–33.71%). This estimate aligns with the incidence of unintended pregnancies in high-income countries (HIC) and middle-income countries (MIC) [31]. However, it was slightly higher than the prevalence reported in a meta-analysis conducted on HIC [29,38]. Nevertheless, the prevalence of unintended pregnancies in developing countries between 2010 and 2014 is much higher (65.5%) [30]. It is important to note that the high prevalence observed in developing countries during this period may be attributed to the inclusion of high-risk populations such as women seeking abortion care services [38].

This umbrella review showed that a lack of communication with the husband regarding family planning and non-use of contraceptives was significantly associated with unintended pregnancy. These findings are consistent with those of previous studies conducted in developing countries [35]. This suggests that a lack of access to family planning and ineffective communication with the husband contribute to an increased risk of unintended pregnancy. Therefore, we recommend providing health education for family planning and for promoting communication with husbands. Additionally, concerned bodies should make efforts to improve the accessibility of family planning services in health care facilities [9,38].

The current review revealed a significant association between unintended pregnancy and higher parity as well as maternal illiteracy. This finding is consistent with those of a systematic review conducted in developing countries [38]. The presence of this association suggests that illiterate mothers lack adequate information on the benefits of family planning and birth spacing. Therefore, it is crucial for programmers, policymakers, and implementers to consider these factors when designing strategies to address unintended pregnancy. Furthermore, the review identified that unmarried women are more likely to experience unintended pregnancies than are married women [38,39]. This can be attributed to the fact that unmarried women may feel ashamed of their sexual activity and may, therefore, refrain from using modern contraceptives. Such risky sexual behaviours can increase vulnerability to unintended pregnancies. These findings highlight the importance of comprehensive sexual education and accessible family-planning services for married and unmarried women. By addressing these factors and promoting open discussions on sexual health, the incidence of unintended pregnancies can be reduced and reproductive health outcomes can be improved [28].

An umbrella review revealed that unintended pregnancy has a significant impact on the lower utilisation of various health care services such as antenatal and institutional care, which is common in LMICs. Several factors contribute to this lower utilisation, including dysfunctional and underperforming health care systems in LMICs, community norms, limited access to transportation and roads, and key socio-demographic factors such as lower socioeconomic conditions, lack of education, and unemployment. These findings are consistent with those of previous study [38,39]. Assessing the opportunities and challenges related to maternal health care service utilisation is crucial for achieving the Sustainable Development Goal (SDG) of universal access to health care services. It is essential to monitor improvements in health care access and identify areas where progress needs to be accelerated, particularly in LMICs where health care service utilisation remains low. By addressing the barriers to health care access, improving health care systems, we can work towards achieving universal access to health care services and improving maternal health outcomes in LMICs [40,41].

In this umbrella review, we discovered that unintended pregnancy increases the risk of maternal depression. This finding aligns with that of a previous study [38]. Additionally, children born to mothers with postnatal depression disorder are more likely to experience stunting than those born to mothers without postnatal depression [9,38]. Another study supports this finding, indicating that children born to depressed mothers are at a higher risk of stunting than are those born from intended pregnancies. Thus, by providing adequate support and interventions for mothers experiencing depression, we can mitigate the negative effects on both maternal well-being and child health outcomes such as stunting [38,40].

Moreover, pregnancy intention and its determinants are related to higher prevalence of risky behaviours during pregnancy, which is known to increase the risk of adverse outcomes [30,39,42,43]. Thus, by addressing comprehensive reproductive health care, including access to contraception, counselling, and support services, we can reduce the incidence of unintended pregnancies and improve maternal and child health outcomes.

### Implications of the study

This study was conducted in response to a previous methodological study in LMICs, which called for a comprehensive synthesis of evidence on unintended pregnancy when multiple systematic reviews exist on the topic. As the first of its kind to synthesise existing systematic reviews and meta-analyses on unintended pregnancies in LMICs, this umbrella review provides a comprehensive summary of this issue. Consequently, the summary estimate of unintended pregnancy, associated factors, and its complications presented in this study can be valuable for clinicians, policymakers, and other stakeholders in optimising maternal and neonatal health in LMICs.

### Strengths and limitations

To minimise bias, rigorous measures were taken, such as comprehensive searches across multiple databases and the involvement of multiple researchers in study selection. The risk of bias in the included systematic reviews and meta-analyses was assessed using AMSTAR software. However, it is important to acknowledge that summarising overlapping primary studies in multiple meta-analyses can potentially lead to overestimation of the findings. Additionally, the nature of meta-analysis, which relies on aggregated data, limits the identification of confounding factors. This may have affected the pooled estimate. Readers should interpret these findings cautiously considering the limitations of both primary studies and umbrella analyses. Moreover, because of the lack of information and data from other regions, generalisation may be challenging.

## CONCLUSIONS

Unintended pregnancy is a significant issue in LMICs, as highlighted by a comprehensive umbrella review. Factors, such as maternal illiteracy, unmarried status, poor communication with the husband, lack of antenatal care, lack of family planning, limited access to delivery care services, place of residence, maternal depression, abortion, stunting, and low birth weight also contribute to this problem. Collaboration among national, international, and local stakeholders is essential to address these challenges. Preventing unintended pregnancies should be a priority, necessitating improved accessibility to maternal and child health care services. Healthcare providers must invest considerable effort into this endeavour.

## Additional material


Online Supplementary Document
